# Management of tooth extraction in a patient with *ELANE* gene mutation-induced cyclic neutropenia: A case report

**DOI:** 10.1097/MD.0000000000017372

**Published:** 2019-09-27

**Authors:** Keiko Aota, Koichi Kani, Tomoko Yamanoi, Yukihiro Momota, Masami Ninomiya, Hiromichi Yumoto, Masayuki Azuma

**Affiliations:** a Department of Oral Medicine; b Department of Periodontology and Endodontology, Tokushima University Graduate School of Biomedical Sciences, Tokushima, Japan.

**Keywords:** cyclic neutropenia, *ELANE* gene, G-CSF, periodontitis, tooth extraction

## Abstract

**Introduction:**

Cyclic neutropenia (CyN) is a rare hematological disease, and patients with CyN often experience an early onset of severe periodontitis and are forced to undergo tooth extraction. Here, we report a case of a patient with CyN who showed different periodicity and oscillations of neutrophil count compared with her mother, despite sharing the same novel genetic mutation.

**Patient concerns:**

A 17-year-old Japanese girl who had been diagnosed with CyN shortly after birth presented to our hospital with a complaint of mobility of her teeth and gingivitis. Upon presentation, an intraoral examination was performed and revealed redness and swelling of the marginal and attached gingiva. Radiographs revealed extreme resorption of the alveolar bone and apical lesions in her mandibular lateral incisors. The patient's hematologic data demonstrated a lack of blood neutrophils (0/μL). The patient had no history of dental extraction, and her mother also had a history of CyN.

**Diagnoses:**

The patient was diagnosed with severe periodontitis that was associated with CyN. Gene testing showed a novel heterozygous mutation in exon 4 of the *ELANE* gene (c.538delC, p.Leu180Ser fsX11).

**Interventions:**

Based on the clinical findings, we planned to extract the patient's mandibular lateral incisors. Although the tooth extraction was scheduled considering the cyclic variation in neutrophil count, the patient's neutrophil count was 0/μL on the day before the planned extraction. Therefore, granulocyte-colony stimulating factor (G-CSF) was administered to increase the patient's neutrophil count. On the day of the patient's admission for the tooth extraction, she presented with fever (body temperature, 38.5°C), tonsillitis, and stomatitis. The extraction was subsequently delayed, and the patient was administered antibiotics and G-CSF for 4 days. At this time, the neutrophil count increased to 750/μL, and the tooth extraction was carried out safely.

**Outcomes:**

The postoperative course was uneventful, and the healing process at the extraction site was excellent.

**Conclusion:**

There is a possibility that the periodicity and oscillations of neutrophil count may change with growth in patients with CyN. Therefore, it is important to frequently examine and treat patients with fluctuating neutrophil levels for the management of invasive dental treatment in patients with CyN.

## 1 Introduction

Cyclic neutropenia (CyN) is a rare hematological disease characterized by periodic neutropenia, with an average 21-day turnover frequency.^[1]^ Neutropenia is defined as the absolute neutrophil count (ANC) and is categorized as mild (ANC: 1000–1500/μL), moderate (ANC: 500–1000/μL), or severe (ANC: <500/μL).^[2]^ Common causes of neutropenia include cancer chemotherapy, autoimmune diseases, drug reaction, and hereditary disorders. The 2 primary genetic forms of neutropenia are CyN and severe congenital neutropenia (SCN).^[3]^ CyN and SCN are usually caused by heterozygous mutations in the gene that encodes neutrophil elastase, *ELANE*.^[4–6]^ CyN is characterized by severe neutropenia (ANC: <0.2 × 10^9^/L) and frequent opportunistic infections, including fever, mouth ulcers, gingivitis, lymphadenopathy, pharyngitis/tonsillitis, and bacterial infections, during the recurrent neutropenic periods.^[7,8]^ In contrast, patients with SCN usually have an ANC <0.5 × 10^9^/L,^[9]^ more serious infections, and may develop acute myeloid leukemia (AML).^[10,11]^

The clinical presentation of CyN was first described in 1910 based on the recurrence of neutropenia, fever, and mouth ulcers in a 19-month-old boy.^[12]^ Although the pathogenesis of the disease has not been completely elucidated, the locus for autosomal dominant cyclic neutropenia is mapped to chromosome 19p13.3, and this disease is attributable to heterozygous mutations of *ELANE.*^[5,6]^ Heterozygous mutations in the *ELANE* gene have been reported in a high frequency of single base pair/amino acid mutations^[13]^ and identified in 80% to 100% of patients with CyN.^[3–6]^ Most laboratory investigations have indicated that mutations in the *ELANE* gene initiate the unfolded protein response, accelerate apoptosis if developing myeloid cells, and result in ineffective myelopoiesis.^[14–17]^

Patients with CyN exhibit oral manifestations, including aphthous ulcers (on fixed or removable mucosa), gingivitis, and periodontitis with alveolar bone loss. These symptoms usually occur around early childhood.^[4]^ Systemic symptoms, such as fever, generally diminish after adolescence, but adults with CyN continue to experience oral ulcers, gingivitis, and periodontitis.^[4,18]^ Thus, oral hygiene management in CyN patients is very important, and infection control should be considered before any invasive dental treatment is performed. Here, we describe the case of a 17-year-old Japanese girl with CyN from whom we were able to successfully extract the mandibular lateral incisors following the administration of recombinant granulocyte-colony stimulating factor (G-CSF) and antibiotics for several days before the extraction. In addition, this patient exhibited a novel heterozygous mutation exon 4 of the *ELANE* gene. Our results demonstrate the importance of managing invasive dental treatment for patients with CyN.

## 2 Case presentation

### 2.1 Presenting concerns

A 17-year-old Japanese girl who had been diagnosed with CyN presented to our hospital with a complaint of mobility of her teeth and gingivitis.

The patient exhibited sepsis at 3 months of age. At 1.5 years of age, bone marrow aspirate showed “maturation arrest” at the promyelocyte or myelocyte stage of neutrophil formation, and she was diagnosed with CyN. From this point forward, the patient had continuously experienced recurrent fevers due to tonsillitis, stomatitis, herpetic stomatitis, and perianal abscesses once a month. Therefore, she had to be hospitalized once or twice per year until she was 15 years of age. Although the recurrence of fever gradually ceased, stomatitis and gingivitis continued to occur, and she developed mobility of the mandibular anterior teeth at 16 years of age.

The patient underwent oral hygiene preventive therapy from early childhood, which was managed by her general dentist. Severe stomatitis frequently interfered with the patient's ability to brush her teeth after meals. At 16 years of age, her general dentist made a fixed bridge on her mandibular anterior teeth due to the congenital deficiency of her mandibular central incisors, and the patient underwent pulpectomy of her mandibular lateral incisors at this time. At 17 years of age, the patient presented to the Department of Periodontology and Endodontology at Tokushima University Hospital because the mobility of her mandibular lateral incisors became severe. The patient's mother also had a history of CyN; however, her father and 2 older brothers were healthy.

### 2.2 Clinical findings

**Figure 1:**
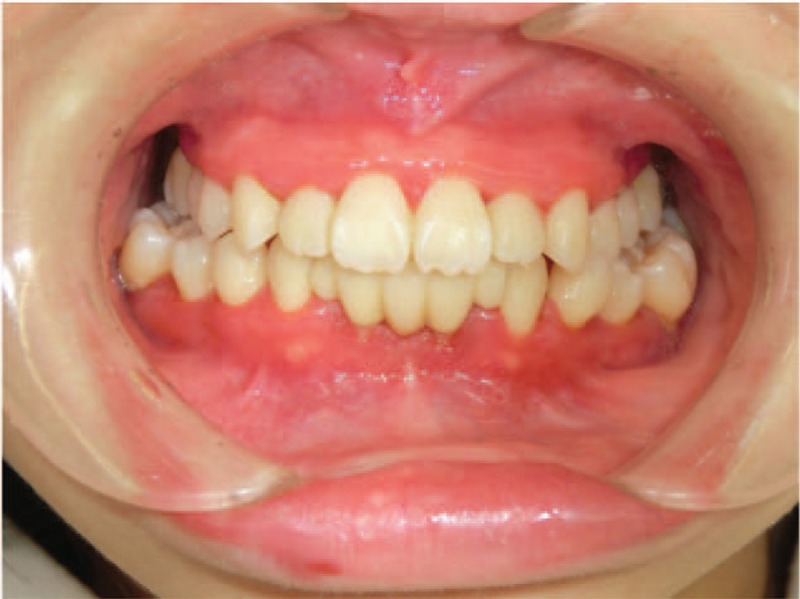
Intraoral view showing redness and swelling of the marginal and attached gingiva.

**Figure 2:**
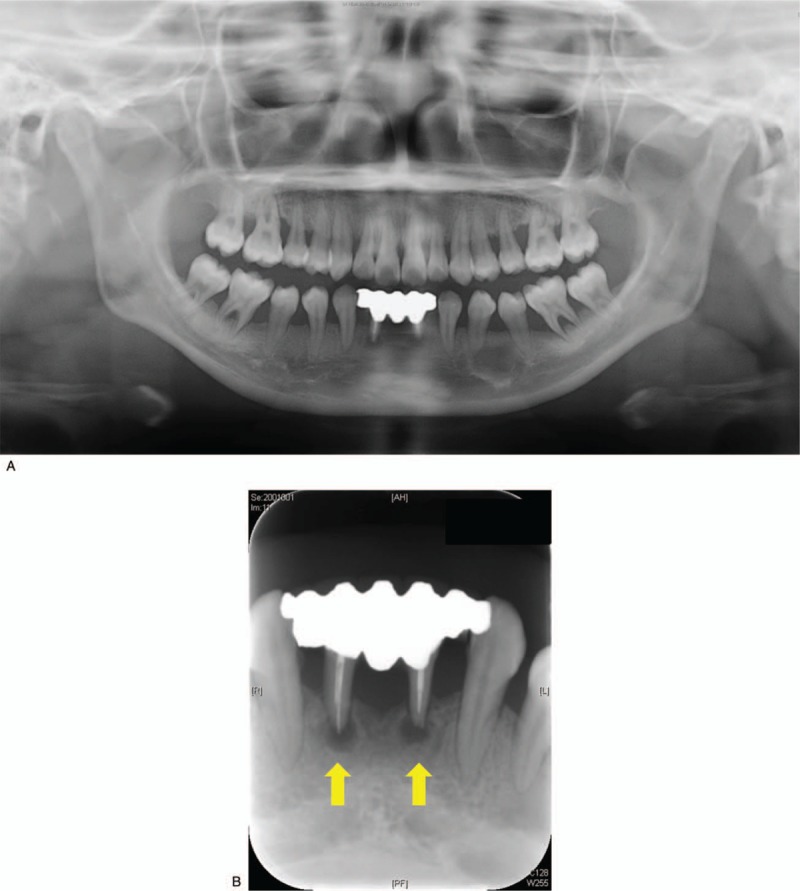
(A) Panoramic radiograph showing extreme resorption of alveolar bone. (B) Dental radiograph demonstrating apical lesions in teeth #32 and #42 (yellow arrows).

An intraoral examination revealed redness and swelling of the marginal and attached gingiva (Fig. 1), a panoramic radiograph showed extreme resorption of the alveolar bone (Fig. 2A), and dental radiographs indicated apical lesions in the mandibular lateral incisors (Fig. 2B). The fixed bridge on the mandibular lateral incisors was attached due to the congenital deficiency of her mandibular central incisors. During the clinical examination, the patient exhibited bleeding on probing (BOP, %) that exceeded 25% and alveolar bone loss that was greater than a three-walled periodontal bone defect and periodontal pockets that exceeded 4 mm around the same teeth; these symptoms were indicative of gingivitis and periodontitis, respectively. Therefore, the patient was diagnosed with CyN-associated gingivitis and periodontitis. We also measured the serum levels of periodontally associated microorganisms. Our patient exhibited significantly higher levels of *Prevotella intermedia*, *Porphyromonas gingivalis*, *Campylobacter rectus*, *Treponema denticola*, and *Tannerella forsythia* than control subjects of the same generation. The patient's periodontal care was initiated by a periodontist, and her mandibular lateral incisors were deemed nonrestorable by the periodontist; therefore, the mandibular lateral incisors needed to be extracted.

### 2.3 Diagnostic assessment

**Figure 3:**
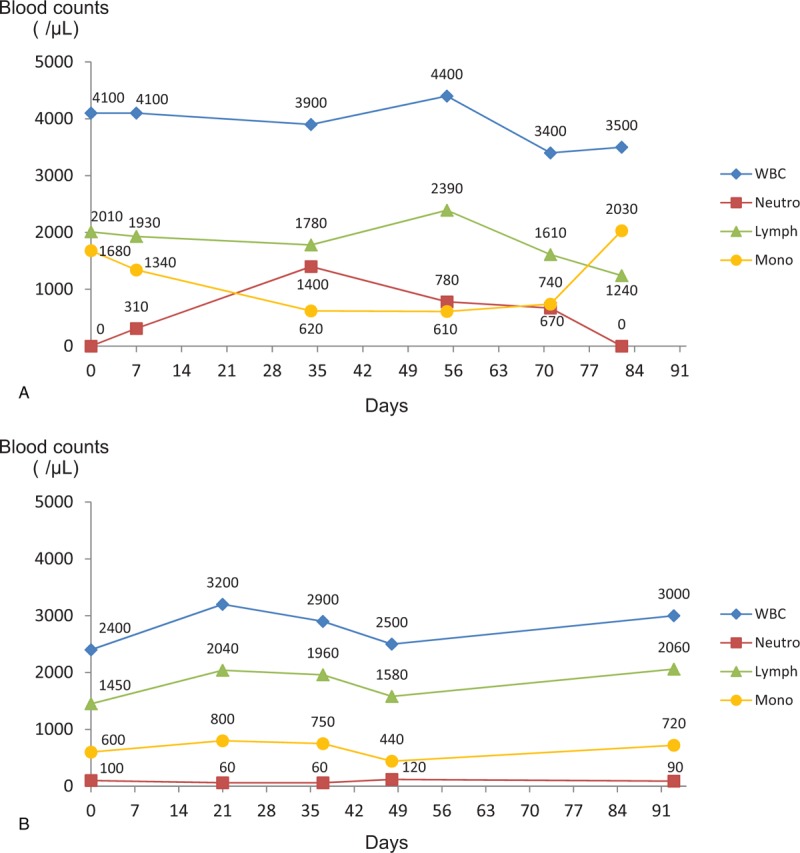
Changes in the WBC, neutrophils, lymphocytes, and monocytes over the course of 3 months. (A) The 17-year-old patient's neutrophil counts appeared to cycle between the range of 0 and 1400/μL, and the monocyte counts show an inverse cycle when compared to the neutrophil counts. (B) The neutrophil counts of the patient's 46-year-old mother did not appear to oscillate, and they ranged between 60 and 120/μL. Lymph = lymphocyte, Mono = monocyte, Neutro = neutrophil, WBC = white blood cell.

The hematologic data on the day of the patient's first visit to our hospital are shown in Table 1. A lack of blood neutrophils (0/μL) and an increased monocyte count (1681/μL) were observed. Total white blood cell (WBC) or leukocyte counts (4100/μL), including lymphocytes (2009/μL), monocytes (1681/μL), eosinophils (307.5/μL), and basophils (102.5/μL), were within normal limits (Table 1). Before tooth extraction, an investigation of the patient's neutropenic cycle, blood testing was conducted over a period of 3 months. As shown in Figure 3A, her neutrophil count was cycled from 0/μL and 1400/μL, whereas the monocyte cycled from 1680/μL to 610/μL (Fig. 3A). In contrast, neutrophil counts of the patient's 46-year-old mother did not appear to oscillate; neutrophils ranged between 60 and 120/μL (Fig. 3B).

**Table 1:**
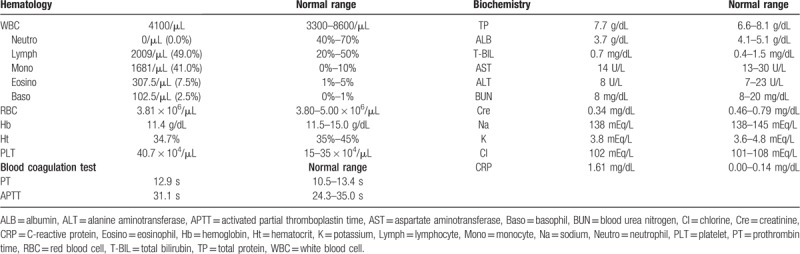
Laboratory data on the day of the patient's first visit.

### 2.4 Therapeutic intervention

Based on the patient's blood test results, we planned to extract her mandibular lateral incisors in the hospital during her spring vacation when her neutrophil count was high. The patient was hospitalized 84 days following her initial visit to our department. However, one day before her admission, the patient's body temperature was 37.4°C and she exhibited erythematous vesicular-bullous lesions (from the second to the third thoracic nerve segments) on the right side of her body. One of our hospital's dermatologists performed a biopsy and detected herpes zoster virus giant cells. The patient was subsequently diagnosed with a herpes zoster infection and treated with an internal medication of amenamevir (Amenalief, Maruho Co., Ltd., Japan) (400 mg/d) for 7 days. On the day of the biopsy, blood tests revealed a WBC count of 3500/μL, neutrophil count of 0/μL (0%), monocyte count of 2030/μL (58.0%), and C-reactive protein (CRP) level of 7.81 mg/dL. Granulocyte-colony stimulating factor (G-CSF) (Lenograstim, Neutrojin, Chugai Pharmaceutical Co., Ltd., Japan) (2 μg/kg) was subcutaneously administered to prevent bacterial infection.

**Figure 4:**
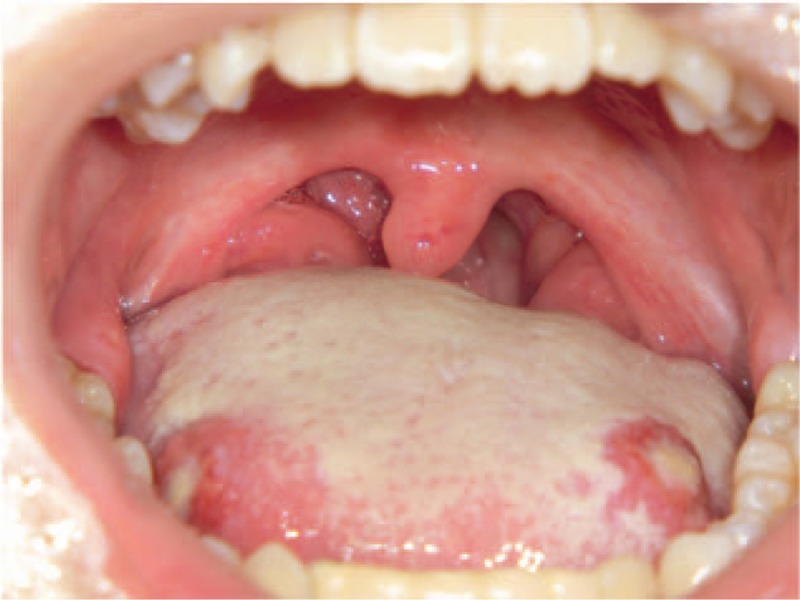
Oral manifestations on admission. A photo of the patient's oral cavity shows tonsillitis and stomatitis.

**Figure 5:**
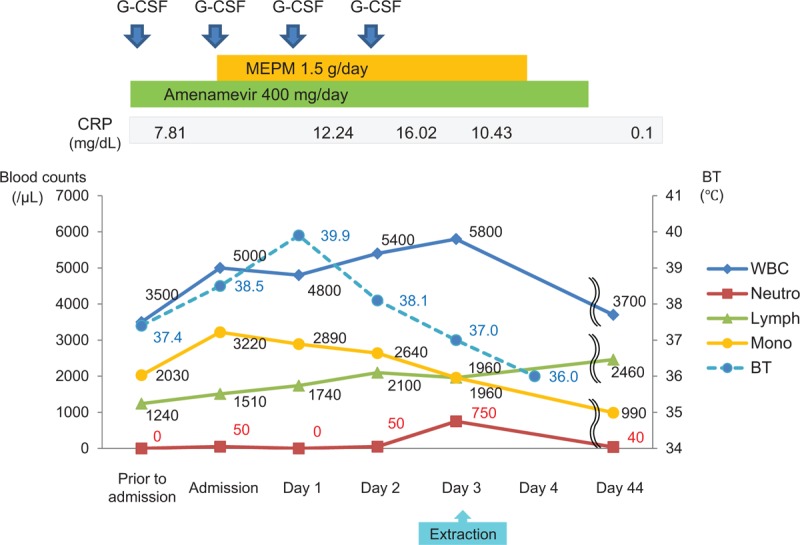
The clinical course after admission. The patient presented with severe neutropenia and an infection with herpes zoster one day before her admission. On the day of admission, she developed a fever that was accompanied by tonsillitis and stomatitis. The clinical symptoms improved after antibiotic and antiviral therapy. The neutrophil count increased to 750/μL after the administration of G-CSF for 4 days. On the fourth day of admission, the extraction of two teeth was carried out safely. BT = body temperature, CRP = C-reactive protein, G-CSF = granulocyte-colony stimulating factor, Lymph = lymphocyte, MEPM = meropenem hydrate, Mono = monocyte, Neutro = neutrophil, WBC = white blood cell.

On the day of the patient's admission for tooth extraction, she developed a fever (body temperature, 38.5°C), which was accompanied by tonsillitis and stomatitis (Fig. 4). The tooth extraction was canceled, and meropenem hydrate (Meropen, Sumitomo Dainippon Pharma Co., Ltd., Japan) (1.5 g/d) was intravenously administered. Because her neutrophil count was still low (50/μL [1%]), a second dose of G-CSF (2 μg/kg) was administered subcutaneously. The following day, the patient's body temperature increased to 39.9°C, and blood tests revealed a WBC count of 4800/μL, neutrophil count of 0/μL (0%), monocyte count of 2890/μL (60%), and CRP level of 12.24 mg/dL. G-CSF treatment was continued daily for 4 days, and therapy with antibiotics was continued daily for 5 days until the tooth extraction was completed. Three days after the patient's admission, her clinical symptoms improved. Her body temperature dropped to 37.0°C, and her neutrophil count increased to 750/μL (13%) (Fig. 5). Based on the patient's improved clinical condition, we decided to perform the tooth extraction.

Local anesthesia was administered into the gingiva around the mandibular lateral incisors using 1.8 mL of 2% lidocaine with 1:80,000 epinephrine. The mandibular lateral incisors were extracted without damaging the gingival soft tissue. The extraction sockets were sutured with 4-0 silk suture. No significant bleeding was observed during or after the procedure. The patient was discharged the next day without fever.

Meropenem hydrate (Meropen) (1.5 g/d) was used as an antibiotic for 4 days before the procedure and one day after the procedure. Cefditoren pivoxil (Meiact MS, Meiji Seika Pharma Co., Ltd., Japan) (300 mg/d) was used for 3 days from the second day after the procedure. In brief, antibiotic was administered for 4 days preoperatively and 4 days postoperatively. The patient did not exhibit postoperative infection, postoperative pain, or delayed healing.

### 2.5 Follow-up and outcomes

A follow-up examination 1 week later revealed excellent healing at the extraction site. The herpes zoster infection had resolved, and no other infections were observed. Temporary teeth were provided and treatment for periodontal disease was continued by her periodontist.

**Figure 6:**
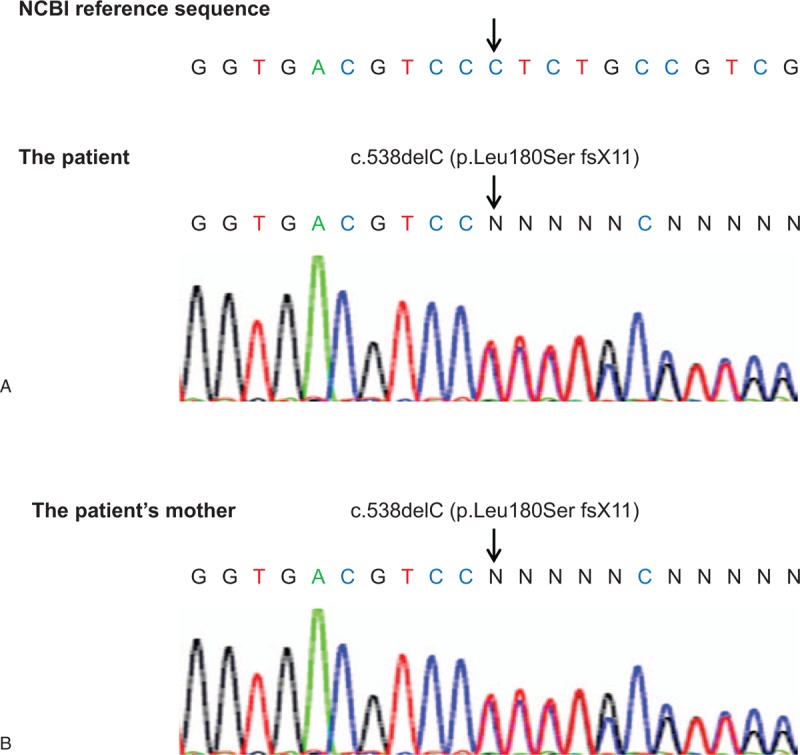
DNA sequence analysis of the *ELANE* gene. Note that both the patient (A) and her mother (B) carry the same mutation (c.538delC, p.Leu180Ser fsX11).

Despite our patient's diagnosis of CyN, based on the clinical course and results of a bone marrow examination, her cycle of neutropenia was not typical and differed from our prediction. The patient and her mother therefore underwent testing for CyN-related genes such as *ELANE* using a polymerase chain reaction analysis and direct sequencing (Hiroshima University). A novel heterozygous sequence variation was noted in exon 4 of the *ELANE* gene (c.538delC, p.Leu180Ser fsX11) (Fig. 6A). The same mutation was detected in the patient's mother (Fig. 6B). This variation was not found in the Human Gene Mutation Database.

## 3 Discussion

Here, we reported the identification of a familial case of CyN resulting from a novel heterozygous mutation in the *ELANE* gene, which encodes neutrophil elastase. This protease is synthesized and packaged in promyelocytes at an early stage in neutrophil development. The etiology of *ELANE*-related neutropenia is attributed to mutations in the *ELANE* gene, which induce unfolded protein response-associated apoptosis at the promyelocyte stage and result in ineffective myelopoiesis.^[14–17]^ Heterozygous mutations in the *ELANE* gene are reported to be found in 80% to 100% of patients with CyN.^[3–6]^*ELANE* heterozygosity has also been identified in 35% to 69% of patients with SCN.^[9,19–23]^ A previous study reported that 187 SCN patients and 120 CyN patients had 94 and 22 mutations in the *ELANE* gene, respectively. In addition, patients with CyN and SCN had 104 distinct *ELANE* mutations.^[13]^ The 104 different *ELANE* mutations comprised 65 missense, 15 frameshift, 8 termination, 8 intronic, 7 in-frame deletions or insertions, and 1 unknown.^[13]^ Most mutations in cases of CyN are observed in exons 4 and 5.^[13]^ Interestingly, the heterozygous variation (p.Leu180Ser missense variant of *ELANE* gene) observed in our patient has not been previously reported in the literature. Therefore, the p.Leu180Ser missense variant of the *ELANE* gene may negatively affect neutrophil elastase function, which plays a pathophysiological role in CyN.

Neutrophils are key immune cells for oral health, and neutrophil deficiency or dysfunction often result in periodontal disease.^[24]^ It has been recognized that patients with CyN often exhibit an early onset of severe periodontitis.^[25–27]^ Conversely, patients with SCN that harbor mutations in the *ELANE* gene present with more severe periodontal diseases as compared with patients with *HAX1* mutations or other unknown mutations.^[18]^ Our patient also presented with severe periodontitis. We measured serum levels of periodontally associated microorganisms and found that the levels of periodontal microorganisms were significantly higher in our patient than control subjects of the same generation. Thus, deficiency in periodontal neutrophils associated with *ELANE* gene mutations may influence the subgingival microbiota composition in the periodontal pocket, leading to the pathogenesis of periodontal breakdown. In addition, the patient could not brush her teeth due to pain during episodes of recurrent stomatitis. Thus, poor oral hygiene may have influenced the development of periodontal disease.

Variations of CyN include fluctuations in neutrophils that can last as long as 3 weeks (probably <5% of individuals) and a reduced amplitude of oscillations.^[4]^ Neutrophil counts in children tend to oscillate more obviously than in adults.^[4]^ The 17-year-old patient's neutrophil cycle appeared to oscillate markedly (range: 0–1400/μL), whereas her 46-year-old mother's cycle showed little oscillation (range: 60–120/μL) despite the same *ELANE* gene mutation. The precise mechanism of oscillations in CyN is not understood, but mutations in the *ELANE* gene are capable of causing the mislocalization of neutrophil elastase and may also induce the unfolded protein response.^[3]^ Neutrophil elastase is produced by the terminally differentiating cohort of neutrophils and ultimately feeds back to inhibit further production of neutrophils, which results in brief interruption of the inhibitory cycle until production of neutrophils resumes, followed again by the inhibitory action of neutrophil elastase in a cyclic manner.^[3]^ Although oscillations in CyN were previously thought to be caused by mutations in the *ELANE* gene, cyclic variations in the neutrophil counts of our patient and her mother were markedly different, although they shared the same *ELANE* mutation. Therefore, the periodicity and oscillations of neutrophil count may involve other factors, such as hormones, in addition to gene mutations.

The perioperative management of patients with CyN requires the prevention and control of infection. Neutropenia is the major cause of surgical site infection (SSI), fever, and sepsis. We discovered only 2 articles with the key words “CyN” and “tooth extraction” that described the cases of a 14-year-old boy and a 15-year-old girl who had been diagnosed with CyN, had periodontal disease, and needed to have their teeth extracted.^[26,28]^ Their invasive dental treatment was performed safely by avoiding the neutropenic cycle and administering antibiotics.

There have been several reports of the perioperative management of invasive dental procedures for patients with neutropenia. Fillmore et al^[29]^ investigated the complications of dental extractions in 116 neutropenic patients. Forty-two percent of the patients showed severe neutropenia (0–499/μL). The main diagnoses associated with neutropenia were myeloid leukemia, lymphoma, lymphoid leukemia, multiple myeloma, pancytopenia or aplastic anemia, myelodysplastic syndrome, and amyloidosis. Most of the patients received preoperative antibiotic coverage; the mean duration of preoperative antibiotic treatment was 2.6 ± 4.85 days (range, 0–30 days), whereas the mean duration of postoperative antibiotic treatment was 4.6 ± 8.78 days (range, 0–60 days). The most commonly used antibiotics were meropenem, cefazolin, clindamycin, cefepime, ampicillin, sulbactam, and vancomycin. Twenty-one patients showed a mean ANC of 691/μL; these patients were administered G-CSF. The overall rate of complications was 8.6% (n = 10), including delayed healing, SSI, and prolonged postoperative pain.

Overholser et al^[30]^ studied dental extractions in 28 patients with acute nonlymphocytic leukemia. Their protocol involved no extraction in patients with severe neutropenia (<500/μL), antibiotic coverage for patients with granulocyte counts <2000/μL, and primary closure of the extraction site. They found no major postoperative complications and 1 patient with SSI.

In the present case, the patient's surgical procedure was scheduled based on a prediction of increased levels of neutrophils. However, contrary to our prediction, the number of neutrophils on the day before her surgery was 0/μL (0%). She additionally developed herpes zoster, tonsillitis, and a fever. The tooth extraction was canceled, and the administration of G-CSF, antibiotics, and antiviral drugs supported the recovery of her general condition. The administration of G-CSF (2 μg/kg) for 4 days was effective in increasing her neutrophil count. The extraction of her teeth and the healing process were successful, and there was no evidence of SSI or fever.

As in this case, the periodicity and oscillations of neutrophil count may change during the course of growth. To avoid complications, it is important to manage invasive dental treatment in patients with CyN by frequently examining neutrophil counts and appropriately treating patients with G-CSF and antibiotics. There are some limitations of the case report or its management.

### 3.1 Patient perspective

Patient Consent Statement: The patient and her mother provided informed consent for publication of the case.

### 3.2 Ethical review and informed consent

This case report was approved by the Ethics Committee of Tokushima University (authorization no. 3236) and the Human Genome/Gene Analysis Research Committee of Tokushima University (authorization no. H30-13). The patient and her mother provided written informed consent.

## Acknowledgments

We thank Dr. Shuhei Karakawa, Department of Pediatrics, Hiroshima University Graduate School of Biomedical & Health Sciences, and Prof. Masao Kobayashi, Department of Pediatrics, Hiroshima University Graduate School of Biomedical & Health Sciences for performing gene testing. This research did not receive any specific grant from funding agencies in the public, commercial, or not-for-profit sectors.

## Author contribution

**Conceptualization:** Keiko Aota.

**Data curation:** Keiko Aota, Masami Ninomiya.

**Investigation:** Keiko Aota, Koichi Kani, Tomoko Yamanoi, Yukihiro Momota, Masami Ninomiya.

**Supervision:** Masayuki Azuma.

**Writing – original draft:** Keiko Aota.

**Writing – review and editing:** Hiromichi Yumoto, Masayuki Azuma.

Keiko Aota orcid: 0000-0001-5413-5750.
